# Do coursework summative assessments predict clinical performance? A systematic review

**DOI:** 10.1186/s12909-017-0878-3

**Published:** 2017-02-16

**Authors:** Rebecca Terry, Wayne Hing, Robin Orr, Nikki Milne

**Affiliations:** Physiotherapy Program, Faculty of Health Sciences and Medicine, Bond University, Gold Coast, 4226 Australia

**Keywords:** Clinical competence, Clinical performance, Workplace performance, Summative assessment, Prediction, Health education, OSCE, Objective Structured Clinical Examination

## Abstract

**Background:**

Two goals of summative assessment in health profession education programs are to ensure the robustness of high stakes decisions such as progression and licensing, and predict future performance. This systematic and critical review aims to investigate the ability of specific modes of summative assessment to predict the clinical performance of health profession education students.

**Methods:**

PubMed, CINAHL, SPORTDiscus, ERIC and EMBASE databases were searched using key terms with articles collected subjected to dedicated inclusion criteria. Rigorous exclusion criteria were applied to ensure a consistent interpretation of ‘summative assessment’ and ‘clinical performance’. Data were extracted using a pre-determined format and papers were critically appraised by two independent reviewers using a modified Downs and Black checklist with level of agreement between reviewers determined through a Kappa analysis.

**Results:**

Of the 4783 studies retrieved from the search strategy, 18 studies were included in the final review. Twelve were from the medical profession and there was one from each of physiotherapy, pharmacy, dietetics, speech pathology, dentistry and dental hygiene. Objective Structured Clinical Examinations featured in 15 papers, written assessments in four and problem based learning evaluations, case based learning evaluations and student portfolios each featured in one paper. Sixteen different measures of clinical performance were used. Two papers were identified as ‘poor’ quality and the remainder categorised as ‘fair’ with an almost perfect (k = 0.852) level of agreement between raters. Objective Structured Clinical Examination scores accounted for 1.4–39.7% of the variance in student performance; multiple choice/extended matching questions and short answer written examinations accounted for 3.2–29.2%; problem based or case based learning evaluations accounted for 4.4–16.6%; and student portfolios accounted for 12.1%.

**Conclusions:**

Objective structured clinical examinations and written examinations consisting of multiple choice/extended matching questions and short answer questions do have significant relationships with the clinical performance of health professional students. However, caution should be applied if using these assessments as predictive measures for clinical performance due to a small body of evidence and large variations in the predictive strength of the relationships identified. Based on the current evidence, the Objective Structured Clinical Examination may be the most appropriate summative assessment for educators to use to identify students that may be at risk of poor performance in a clinical workplace environment. Further research on this topic is needed to improve the strength of the predictive relationship.

**Electronic supplementary material:**

The online version of this article (doi:10.1186/s12909-017-0878-3) contains supplementary material, which is available to authorized users.

## Background

Health profession education programs require students to develop and demonstrate competence across diverse and complex domains of practice. The curriculums delivered across the medical, nursing and allied health professions vary in the attitudes, knowledge and skills required of their graduates. However, there are many similarities in the domains of competence required by the registration bodies of these professions. To be a licenced medical, nursing or allied health professional, graduates must demonstrate competence across domains of practice such as: professional and ethical behaviour, communication and interpersonal skills, knowledge, safety and quality, leadership and management, and collaborative practice [1–3]. Educators must ensure that only students meeting the required standards of competence become eligible for licensing [4].

As the domains of practice required by the different health professions share similarities, so to do the assessment frameworks used by their education programs [5]. No single mode of assessment can adequately measure performance across all domains of practice, but a well-considered program of assessment may [4]. Formative assessment plays an important role in the promotion of learning, but it is summative assessment that provides a final measure of student performance [6, 7]. Summative assessment in health profession education has three main goals: (i) the promotion of future learning, (ii) to ensure that high-stakes decisions such as progression, graduation and licensing are robust so the public is protected from incompetent practitioners, (iii) and to provide a basis for choosing applicants for advanced training [8]. To achieve the goals of providing robust evidence of competence, and the identification of appropriateness for advance training, summative assessments scores must necessarily be predictive of student’s future performance. However, there is limited evidence to support this assumption.

A systematic review by Hamdy et al. [9] of predictors of future clinical performance in medical students found OSCEs and pre-clinical grade point average (GPA) to be significant predictor variables for clinical performance, however the predictive relationships were limited. Additionally, a compilation and review of correlative studies by Harfmann and Zirwas [10] looked to answer whether performance in medical school could predict performance in residency. In their review, medical student pre-clinical GPA scores were one of the indicators that correlated most strongly with performance on examinations in residency.

While the reviews by Hamdy [9] and Harfmann and Zirwas [10] looked at a range of predictor variables, the only specific mode of summative assessment common to all health professions evaluated was the Objective Structured Clinical Examination (OSCE) and this was limited only to medical education programs. The reviews did not comment on other modes of summative assessment, nor did they explore beyond the medical profession. On this basis, the ability of a variety of modes of assessment to predict future clinical performance has yet to be investigated in detail.

The aim of this review was to critically appraise and discuss the findings of existing research investigating modes of summative assessment, and their ability to predict future clinical performance. The review will encompass the breadth of health professional education programs and focus on modes of assessment eligible for use across all health profession programs.

## Methods

### Search strategy

Peer reviewed research papers were gathered using a search of the PubMed, CINAHL, SPORTDiscus, ERIC and EMBASE databases. Key search terms were chosen to capture the breadth of assessments commonly used within the non-clinical components of health profession programs, as well as the variety of terms used to describe performance in a clinical setting. These search terms were generated following consultation with educators from health professions and are outlined in Table 1.

**Table 1:** Systematic review databases and search terms

Database	Search Terms	
PubMed CINAHL SPORTDiscus EMBASE ERIC		student*
	AND	predict* OR associat* OR correlat* OR relat*
	AND	clinical performance OR clinical practice OR work* performance OR
	AND	summative assess* OR OSCE OR objective structured clinical examination OR practical exam* OR practical assess* OR written exam* OR written assess* OR theory exam* OR theory assess* OR oral exam* OR oral assess* OR oral presentation OR VIVA OR viva voce OR clinical exam* OR clinical assess* OR

### Screening and selection

Title and abstracts of all papers identified by the initial database searches were screened and assessed against the following inclusion criteria:

- The paper reported on the relationship between assessment results and the future clinical performance of students in health professional programs; and
- The paper was published in the English language; and
- The paper was published after 1996.

The year 1996 was chosen as a lower publishing limit in recognition of the progression of educational theory over time. This date allows for the capture of 20 years of literature following on from the seminal papers by Harden [11] regarding the development of the OSCE and Miller’s framework for the assessment of clinical competence [12].

Papers selected for inclusion from the initial database searches were then subject to the application of rigorous exclusion criteria:

- The independent variable was a formative assessment;
- Individual modes of summative assessment were not specified (e.g. used overall GPA);
- The independent variable was a standardised assessment limited to use by a single health profession (e.g. National Board of Medical Examiners subject examinations);
- The independent variables were health profession education program admission criteria, applicant screening measures or entry measures;
- Clinical performance was not measured in either a clinical workplace setting or in a clinical examination conducted externally to the education program utilizing authentic or standardized patients; or
- The paper was an abstract, review, dissertation or discussion

**Fig. 1:**
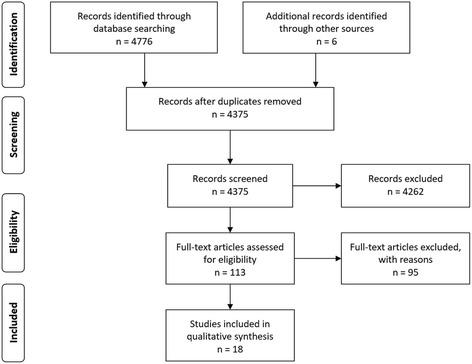
Preferred reporting items for systematic reviews and meta-analyses (PRISMA)

The exclusion criteria listed above were applied to ensure reasonable consistency between papers in the interpretation of ‘summative assessment’ and ‘clinical performance’ to allow for a cohesive synthesis of the information. Review papers were used to provide background and supporting information. To ensure maximal search saturation a secondary search of the reference lists of papers retained for review, and papers providing background or supporting information were scanned for potentially relevant articles. These articles were then gathered and subjected to the same inclusion and exclusion criteria described above (Fig. 1).

### Critical appraisal of methodological quality

Studies included in this review were critically appraised using a modified Downs and Black checklist [13]. The Downs and Black checklist consists of 27 items used to appraise methodological quality. The checklist was originally devised to assess the methodological quality of health care interventions, however it was appropriate to use in this review as it provided a structured format for critically appraising the papers selected for review. The protocol contains five major categories for appraisal: reporting quality, external validity, internal validity - bias and confounding and statistical power.

The original Downs and Black checklist is scored out of 32. All items excepting Items 5 and 27 are scored on a two-point scale. A classification as ‘yes’ is scored as ‘1’ point and a classification of ‘no’ or ‘unable to determine’ is scored as ‘0’ points. Item 5, which appraises the description of confounders is scored out of ‘2’ points, with ‘yes’ scoring ‘2’ points, a ‘partial description’ scoring ‘1’ point and ‘no’ scoring ‘0’ points. Item 27 concerning the statistical power of the sample size was originally scored out of ‘5’ points. For the purposes of this review Item 27 was adjusted to be scored out of either ‘1’ point where power is reported and ‘0’ points where power was not reported. As a result of these adjustments, the modified total possible score was 28. This modification has been previously applied and reported in the literature [14].

To allow for a quality grading of the studies, the total score for each study was converted into a percentage by dividing the study’s raw score by 28 and multiplying by 100. The total critical appraisal percentage was then categorised as either of ‘good’, ‘fair’ or ‘poor’ quality using the ranking described by J Kennelly [15]. When applied to the modified Downs and Black scoring Kennelly’s model categorises papers with critcal appraisal scores of 71% or greater as good quality, 54-70% as fair quality and 53% or less as poor quality.

Each paper was individually rated by two assessors (RT and NM) with the level of agreement determined by a Kappa analysis conducted by a third person (RO). Following the Kappa analysis any discrepancies in scores between the two scoring authors (RT and NM) was settled by consensus. Where consensus could not be reached, the raw scores were adjudicated by a third person (RO) to finalise the Critical Appraisal Score (CAS).

### Data extraction and synthesis

Data from each paper included in the review were extracted by a single author (RT) and confirmed by the fellow authors. Data were assessed using a pre-determined format as follows: clinical education program, number of students, student year of study, summative assessments used, clinical setting in which performance was measured and statistics used to establish relationships. Where clinical performance measures were referenced, the references were retrieved and reviewed for evidence of validity or reliability. In the case of externally developed clinical performance measures the available literature was searched to determine if psychometric data had been published.

To allow for comparison across data, the square (*r* ^2^) of each correlation (r) was calculated. Squaring the correlation gives the variance which measures the proportion of variability in one variable that is explained by the relationship with the other variable [16]. In this review, the variance describes the proportion of variability in student’s clinical performance explained by summative assessment scores.

## Results

### Literature search and selection

The results of the search are reported in Fig. 1. After the application of inclusion and exclusion criteria 18 papers were retained for final analysis. Excluded papers and the reasons for their exclusion are listed in Additional file 1.

### Study participants

The papers retained for the final review reported on summative coursework assessments and student performance in the clinical setting and are summarised in Table 2. Across these papers seven different clinical professions were represented: medicine or osteopathic medicine (12), pharmacy (1), physiotherapy (1), dietetics (1), speech pathology (1), dentistry (1) and dental hygiene (1). Student populations studied were from the United States of America (11), Australia (2), Canada (1), the United Kingdom (1), New Zealand (1), South Korea (1) and Hong Kong (1).

**Table 2:** Summary of critical review papers

*Author and Population*	*n*	*Summative Assessments*	*Clinical Setting*	*Clinical Performance Measure* (*CPM*)	*Evidence CPM has Validity or Reliability*
*Baker*, *Cope* et al. [25] *Osteopathic Medicine*, *USA*	*70*	• *Year 3 OSCE*	*COMLEX*-*USA Level 2* -*PE*	*COMLEX*-*USA Level 2*-*PE* (*Pass or fail*)	*Y* [40, 41]
*Berg* et al. [23] *Medicine*, *USA*	*217*	• *Year 3 OSCE* - *Data Gathering*, *Documentation and Communication*/*Interpersonal Skills subsets*	*USMLE Step 2 CS*	*USMLE Step 2 CS* - *Data Gathering*, *Documentation & Communication*/*Interpersonal skills subsets*	*Y* [42]
*Campos*-*Outcalt* et al. [30] *Medicine*, *USA*	*308*	• *4th Year OSCE*	*First year of residency training* – *environment not specified*	*Residency director ratings*	*N*
*Carr* et al. [20] *Medicine*, *Australia*	*200*	• *Year 4 and 5 OSCE* • *Year 5 Written Examination* – *5 Essay and 5 SAQ* • *Year 6 Written Examination* – *100 EMQ and 10 SAQ*	*Tertiary hospitals*	*Junior Doctor Assessment Tool* (*JDAT*)	*Y* [43]
*Cope*, *Baker* et al. [26] *Osteopathic Medicine*, *USA*	*70*	• *Year 3 OSCE*	*Clinical rotations* – *environment not specified*	*Clinical Education Grade Form*	*N*
*Dong* et al.[24] *Medicine*, *USA*	*806*	• *Year 2 & 3 OSCE*	*USMLE Step 2 CS*	*USMLE Step 2 CS* – *Integrated Clinical Encounter* (*ICE*) *and Communication and Interpersonal Skills* (*CIS*) *component*	*Y* [42]
*Ferguson* et al. [19] *Medicine*, *USA*	*157*	• *Year 2 MCQ Examination* • *Second semester case based learning evaluations* - *Group participation* - *Written reports*	*Third year clerkships*	*Clinical evaluation form*	*Y* [44]
*Gadbury*-*Amyot* et al. [18] *Dental Hygiene*, *USA*	*74*	• *Portfolio*	*Central Region Dental Testing Service* (*CRTDS*)	*CRTDS clinical licensure examination*	*Y* [45]
*Graham* et al. [34] *Dentistry*, *USA*	*145*	• *OSCE*	*Third year clinical training* – *environment not specified*	*Clinical productivity value* – *combined score for successfully completed clinical procedures*	*N*
*Han* et al. [27] *Medicine*, *South Korea*	*63*	• *OSCE* • *Clinical Performance Examination*	*Internship* – *Chonnam National University Hospital*	*Intern performance scores* – *5*-*pt Likert scale*	*N*
*Hawker* et al. [32] *Dietetics*, *Australia*	*193*	• *OSCE*	*7 week clinical placements* – *environment not specified*	*Clinical teacher assessments with standardised rubric*	*N*
*Ho* et al. [17] *Speech Pathology*, *Hong Kong*	*71*	• *PBL Evaluations* – ○ *Reading form* ○ *Reflective journal* ○ *Tutorial process assessments*	*Year 2* – *In*-*house clinic* *Year 3* – *Hospitals and community rehabilitation centers*	*COMPASS*® *Hong Kong University Clinical Forms*	*Y* [46, 47] *N*
*Kahn* et al. [29] *Medicine*, *USA*	*50*	• *OSCE*	*First year of residency training* – *environment not specified*	*Residency program director overall evaluations* – *5*-*pt Likert Scale*	*N*
*LaRochelle* et al. [22] *Medicine*, *USA*	*514*	• *OSCE* • *Written examinations* – ×*2 MCQ and ×1 cumulative essay*	*First year of residency training* – *environment not specified*	*Program director evaluation form* (*PGY*-*1 PD*)	*Y* [48]
*McLaughlin* et al. [33] *Pharmacy*, *USA*	*289*	• *Year 2* (*Spring and Fall*) *OSCE and Year 3* (*Fall*) *OSCE*	*Year 4 Advanced Pharmacy Practice Experiences*	*Online evaluation form*	*N*
*Probert* et al. [28] *Medicine*, *UK*	*30*	• *OSCE*	*Hospitals 1 year after graduation*	*Senior doctor assessments* – *5*-*pt Likert scale*	*N*
*Wessel* et al. [31] *Physiotherapy*, *Canada*	*48*	• *OSCE*	*6*-*week clinical practicum* – *environment not specified*	*Physiotherapy Clinical Performance Instrument* (*PT CPI*)	*Y* [49, 50]
*Wilkinson & Frampton* [21] *Medicine*, *New Zealand*	*117*	• *OSCE* • *Written 1*– *3* × *3 h short and long essay* • *Written 2* – *1* × *3 h short essay and 2* × *3 h EMQ*	*Trainee internship year*	*Global rating instrument*	*Y* [51]

*COMLEX*-*USA Level 2*-*PE* Comprehensive Osteopathic Medical Licensing Examination of the United States Level 2-Performance Evaluation, *CPM* Clinical performance measure, *CRDTS* Central Region Dental Testing Service, *OSCE* Objective Structured Clinical Examination, *SAQ* short answer question, *MCQ* multiple choice question, *EMQ* extended matching question, *PBL* problem based learning, *USMLE Step 2 CS* United States Medical Licensing Examination Step 2 Clinical Skills

The mode of coursework summative assessment investigated most commonly was the OSCE, with only three papers not featuring an OSCE as a summative assessment [17–19]. Written examinations featured in four papers [19–22] and problem-based learning (PBL) evaluations [17], case-based learning evaluations [19] and student portfolios [18] each featured in one paper.

Measures of clinical performance used in the medical programs were: the United States Medical Licensing Examination Step 2 Clinical Skills (USMLE Step 2 CS) [23, 24]; the Comprehensive Osteopathic Medical Licensing Examination of the United States Level 2-Performance Evaluation (COMLEX-USA Level 2-PE) [25]; a Clinical Education Grade Form [26]; a standardised Clinical Evaluation Form [19]; intern performance scores [27]; senior doctor assessments [28]; the Junior Doctor Assessment Tool (JDAT) [20]; a global rating instrument [21]; program director evaluations [22] and residency program director assessments [29, 30]. A variety of clinical performance measures were used amongst the allied health programs: the Physiotherapy Clinical Performance Instrument (PT CPI) [31]; the National Dental Hygiene Examination (NDHE) [18]; the Hong Kong University (HKU) speech pathology clinical evaluation form and COMPASS®: Competency Based Assessment in Speech Pathology [17]; a standardized dietetics clinical teacher evaluation rubric [32]; an online evaluation form of pharmacy student performance [33] and a dental clinical productivity value [34].

### Critical appraisal of methodological quality

Percentage scores based on the modified Downs and Black [13] checklist ranged from 29% [19] to 68% [21] with a mean percentage of 56.15% (±8.29%). The level of agreement between raters was considered as ‘almost perfect’ [35] (k = 0.852). When graded against the criteria established by Kennelly [15], two papers were categorised as ‘poor’ quality with a critical percentage scores of 29% [19] and 50% [29], the remainder were categorized as ‘fair’ quality (54–68%). All of the studies included in the review were descriptive cohort studies.

Analysis of the mean and standard deviations of the categories of the modified Downs and Black checklist were conducted and showed the mean score achieved in the ‘*reporting*’ category to be 5.94 points (±1.35 points) out of a possible 11 points. Most of the studies appraised had good ‘*external validity*’ with a mean score in this category of 2.5/3 points. The mean score in the ‘*internal validity* – *bias*’ category was 4.33 points (±0.69 points) out of a possible 7 points. Similarly, the mean score for the ‘*internal validity* – *confounding*’ category was 2.94 points (±0.85 points) out of a possible 6 points.

The critical review findings are displayed in Table 3. All but four papers [22, 28, 32, 34] used either Pearson’s correlation, Spearman’s rho or point-biserial correlations to identify the relationship between summative assessment scores and clinical performance ratings. One paper reported correlations but did not specify the type [26]. Variances are listed in Table 4 and ranged from 1.4 to 39.7%.

**Table 3:** Critical Review Findings

*Author and Population*	*Statistic*	*Findings*	*CAP*
*Baker*, *Cope* et al. [25] *Osteopathic Medicine*	• *Point biseral correlations*	*Significant* (*p* < *0.01*) *correlation between pass*/*failure of COMLEX*-*USA Level 2*-*PE and OSCE*:	*64* % *Fair*
		• *Total OSCE score r* = *0.33*	
		• *Physical examination subscore r* = *0.40*	
*Berg* et al. [23] *Medicine*	• *Pearson*’*s correlation*	*Significant* (*p* < *0.05*) *correlations between the same subsets across tests*.	*54* % *Fair*
		• *Data gathering r* = *0.18*	
		• *Documentation r* = *0.35*	
		• *Communication*/*personal r* = *0.32*	
*Campos*-*Outcalt* et al. [30] *Medicine*	• *Pearson*’*s correlation*	*Significant* (*p* < *0.01*) *correlations between residency director ratings and OSCE*:	*57* % *Fair*
		• *Total OSCE score r* = *0.305*	
*Carr* et al. [20] *Medicine*	• *Pearson*’*s correlation* • *Linear regression with Bonferroni adjustment*	*Significant correlations between the overall JDAT Score and the*:	*64* % *Fair*
		*Year 6 Written r* = *0.178*, *p* = *0.014*	
		*Year 4 OSCE r* = *0.137*, *p* = *0.027*	
		*Year 5 OSCE r* = *0.161*, *p* = *0.022*	
		*Linear regression model found individual summative assessments did not demonstrate a significant influence on overall JDAT score* (*p*-*values of 0.141*–*0.859*).	
*Cope*, *Baker* et al. [26] *Osteopathic medicine*	• *Correlations*	*Significant* (*p* < 0.05*; *p* < 0.01**) *correlations between subscores of the Clinical Evaluation Grade Form and OSCE measures*:	*54* % *Fair*
		OSCE Total and Subscores 1**, 2*, *3*–5** *r* = *0.25*–*0.43*	
		*History taking and Subscores 1*,*3*–5** *r* = *0.31*–*0.40*	
		*Physical Examination and Subscores 1*,*3*,5* *r* = *0.24*–*0.29*	
		SOAP Note Form and Subscores 1**, 2*, 3**, 5* *r* = *0.28*–*0.34*	
*Dong* et al. [24] *Medicine*	• *Pearson*’*s correlation*	*Significant correlations between USMLE Step 2 CS components and OSCEs*.	*57* % *Fair*
		*Year 2 OSCE and Integrated Clinical Encounter Component r* = *0.25*	
		*Year 2 OSCE and Communication and Interpersonal Skills Component r* = *0.26*	
		*Year 3 OSCE and Integrated Clinical Encounter Component r* = *0.16*	
		*Year 3 OSCE and Communication and Interpersonal Skills Component r* = *0.27*	
*Ferguson* et al. [19] *Medicine*	• *Pearson*’*s correlation*	*Significant correlations between clinical evaluation form and*:	*29* % *Poor*
		*MCQ Written examination r* = *0.27*, *p* = *0.0009*	
		*Case based learning reports*	
		–*Group participation r* = *0.28*, *p* = *0.0004*	
		- *Written reports r* = *0.21*, *p* = *0.009*	
*Gadbury*-*Amyot* et al. [18] *Dental Hygiene*	• *Pearson*’*s correlation* • *Linear regression*	*Significant* (*p* < *0.05*) *correlation between Portfolio total score and CRDTS score*	*54* % *Fair*
		*r* = *0.27*	
		*A prediction model using two factors predicted 13.9* % *of the variance in Central Region Dental Service Testing scores*	
*Graham* et al. [34] *Dentistry*	• *Polynomial regression*	*Significant* (*p* < *0.001*) *correlation between OSCE and clinical productivity value*	*61* % *Fair*
		*2010 Cohort r* = *0.614*	
		*2011 Cohort r* = *0.54*	
*Han* et al. [27] *Medicine*	• *Pearson*’*s correlation*	*Significant correlation between mean intern performance scores and OSCE*	*57* % *Fair*
		*r* = *0.278*, *p* < *0.028*	
		*Significant correlation between mean intern performance and CPX subsets*	
		*Patient*-*physician interaction r* = *0.503*, *p* < *0.001*	
		*Clinical skills r* = *0.278*, *p* < *0.027*	
*Hawker* et al. [32] *Dietetics*	• *Linear regression*	*Identified a* β *coefficient of 0.66* (*p* <*0.0001*) *between individual OSCE scores and placement scores*	*61* % *Fair*
*Ho* et al. [17] *Speech Pathology*	• *Spearman*’*s rho*	*Significant correlations* (*p* < 0.01**; *p* < 0.05*) *between*:	*54* % *Fair*
		*treatment skills and interpersonal skill subsets of the HKU clinical form and*	
		*Reflective journal r* = 0.331**, 0.272*	
		*Tutorial process r* = 0.242*, 0.280*	
		*COMPASS*® *generic competencies and tutorial process r* = *0.315*–0.407**	
		*COMPASS*® *overall occupational competency scores and*	
		*Reflective journal r* = 0.271*	
		*Tutorial process r* = 0.367**	
*Kahn* et al. [29] *Medicine*	• *Pearson*’*s correlations* • *Spearman*’*s rho*	*No significant correlations between OSCE and program director overall evaluations*.	*50* % *Poor*
		*r* = *0.22*, *p* = *0.15*	
*LaRochelle* et al. [22] *Medicine*	• *Multiple linear regression*	*The OSCE was a significant predictor of PGY1*-*PD Medical Expertise scores in a model containing multiple independent variables* (β = *0.134*, *p* = *0.013*). *The written examination were not significant predictors of PGY1*-*PD scores*, *although approached statistical significance* (β = *0.266*, *p* = *0.07*).	*54* % *Fair*
		*The OSCE was the only significant predictor of PGYI*-*PD Professionalism scores in a model containing multiple independent variables* (β = *0.124*, *p* < *0.026*)	
*McLaughlin* et al. [33] *Pharmacy*	• *Pearson*’*s correlations*	*Significant* (*p* < 0.05*; *p* < 0.01**) *correlations between OSCEs and specific APPEs*: *acute care*, *ambulatory care*, *clinical specialty and community*	*57* % *Fair*
		*Year 2 Fall OSCE and all four APPEs r* = 0.13*–0.14*	
		*Year 2 Spring OSCE and acute care APPE r* = 0.12*	
		*Year 3 Fall OSCE and*:	
		*acute care APPE r* = 0.12*	
		*ambulatory care APPE r* = 0.25**	
		*clinical specialty APPE r* = 0.13*	
*Probert* et al. [28] *Medicine*	• *Logistic regression*	*No statistically significant results*.	*57* % *Fair*
		*OR 1.64*, *95* % *CI 0.50*–*5.41*	
		*OSCE showed trend of positive association with senior doctor assessments*.	
*Wessel* et al. [31] *Physiotherapy*	• *Spearman*’*s rank correlations*	*No significant correlations between OSCE average score and Physiotherapy Clinical Performance Instrument average score*.	*61* % *Fair*
*Wilkinson & Frampton* [21] *Medicine*	• *Pearson*’*s correlation*	*Significant* (*p* < 0.01*, *p* < 0.001**) *correlations between global rating instrument*:	*68* % *Fair*
		*Total score and*: *OSCE r* = 0.59**	
		*Written 2 r* = 0.54**	
		*Clinical skills subset and*: *OSCE r* = 0.63**	
		*Written 2 r* = 0.57**	
		*Humanistic subset and*: *OSCE r* = 0.44**	
		*Written 2 r* = 0.41*	

*APPE* Advanced Pharmacy Practice Experiences, *CAP* Critical appraisal percentage, *COMLEX* *−USA Level 2-PE* Comprehensive Osteopathic Medical Licensing Examination of the United States Level 2-Performance Evaluation, *CPX* clinical performance examination, *HKU* Hong Kong University, *JDAT* Junior Doctor Assessment Tool, *OSCE* objective structured clinical examination, *PGY*-*1 PD* program director evaluation form, *USMLE Step 2 CS* United States Medical Licensing Examination Step 2 Clinical Skills

**Table 4:** Proportion of variability accounted for by the relationship between summative assessment and clinical performance

Study	Relationship	Correlation (r)	*p*- value	Variance (*r* ^2^)	%
*Baker* et al. [25] *Osteopathic Medicine*	*OSCE measures and COMLEX*-*USA Level 2*-*PE Pass or Failure*				
	*OSCE Total score*	*0.33*	<*0.01*	*0.109*	*10.9* %
	*OSCE Physical Examination subscore*	*0.40*	<*0.01*	*0.16*	*16*.0 %
*Berg* et al. [23] *Medicine*	*OSCE and USMLE Step 2 CS Data Gathering*	*0.18*	<*0.05*	*0.032*	*3.2* %
	*OSCE and USMLE Step 2 CS Documentation*	*0.35*	<*0.05*	*0.123*	*12.3* %
	*OSCE and USMLE Step 2 CS and Communication*/*Personal*	*0.32*	<*0.05*	*0.102*	*10.2* %
*Campos*-*Outcalt* et al. [30]	*OSCE total score and residency director ratings*	*0.305*	<*0.01*	*0.093*	*9.3* %
*Carr* et al. [20] *Medicine*	*Year 5 Written exam* (*5 modified essay questions* + *5 SAQ*) *and JDAT overall score*	*0.076*	*0.148*	*0.022*	*2.2* %
	*Year 6 Written exam* (*100 EMQ* + *10 SAQ*) *and JDAT overall score*	*0.178*	*0.014*	*0.032*	*3.2* %
	*Year 4 OSCE and JDAT overall score*	*0.137*	*0.027*	*0.019*	*1.9* %
	*Year 5 OSCE and JDAT overall score*	*0.161*	*0.022*	*0.026*	*2.6* %
*Cope*, *Baker* et al.[26] *Osteopathic medicine*	*OSCE measures and Clinical Evaluation Grade Form subscores*				
	*OSCE Total and Subscores 1*, *3*–*5*	*0.31*–*0.43*	<*0.01*	*0.096*–*0.185*	*9.6*–*18.5* %
	*OSCE Total and Subscore 2*	*0.25*	<*0.05*	*0.063*	*6.3* %
	*OSCE* - *History score and Subscores 1*, *3*–*5*	*0.31*–*0.40*	<*0.01*	*0.096*–*0.16*	*9.6*–*16* %
	*OSCE* - *Physical Examination score and Subscores 1*,*3*,*5*	*0.24*–*0.29*	<*0.05*	*0.058*–*0.084*	*5.8*–*8.4* %
	*OSCE* - *SOAP Note Form score and Subscore 1*, *3*	*0.34*–*0.38*	<*0.01*	*0.116*–*0.144*	*11.6*–*14.4* %
	*OSCE* - *SOAP Note Form score and Subscores 2*,*5*	*0.28*–*0.30*	<*0.05*	*0.078*–*0.090*	*7.8*–*9* %
*Dong* et al. [24] *Medicine*	*Year 2 OSCE and USMLE Step 2 CS ICE Component*	*0.25*	<*0.01*	*0.063*	*6.3* %
	*Year 2 OSCE and USMLE Step 2 CS CIS Component*	*0.26*	<*0.01*	*0.068*	*6.8* %
	*Year 3 OSCE and USMLE Step 2 CS ICE Component*	*0.16*	<*0.01*	*0.026*	*2.6* %
	*Year 3 OSCE and USMLE Step 2 CS CIS Component*	*0.27*	<*0.01*	*0.073*	*7.3* %
*Ferguson* et al. [19] *Medicine*	*MCQ Written Examination and Clinical Evaluation Form*	*0.27*	*0.0009*	*0.073*	*7.3* %
	*Case based learning measures and Clinical Evaluation Form*				
	*Case based learning group participation*	*0.28*	*0.0004*	*0.078*	*7.8* %
	*Case based learning written reports*	*0.21*	*0.009*	*0.044*	*4.4* %
*Gadbury*-*Amyot* et al. [18] *Dental Hygiene*	*Portfolio and CRDTS clinical licensure examination*	*0.27*	<*0.05*	*0.073*	*7.3* %
*Graham* et al. [34] *Dentistry*	*OSCE and Clinical Productivity Value*	*2010 Cohort* = *0.614*	<*0.0001*	*0.377*	*37.7* %* - 29.2* %
		*2011 Cohort* = *0.54*	<*0.0001*	*0.292*	
*Han* et al. [27] *Medicine*	*OSCE and Intern performance score*	*0.278*	*0.028*	*0.077*	*7.7* %
	*CPX clinical skills subset and intern performance score*	*0.278*	*0.027*	*0.077*	*7.7* %
	*CPX patient*-*physician interaction subset and intern performance score*	*0.503*	<*0.001*	*0.253*	*25.3* %
*Ho* et al. [17] *Speech Pathology*	*PBL Evaluation Reflective Journal and HKU clinical form*				
	- *Treatment skill subset*	*0.331*	<*0.01*	*0.110*	*11.0* %
	- *Interpersonal skill subset*	*0.272*	<*0.05*	*0.074*	*7.4* %
	*PBL Evaluation Tutorial Process and HKU clinical form*				
	- *Treatment skill subset*	*0.242*	<*0.05*	*0.059*	*5.9* %
	- *Interpersonal skill subset*	*0.280*	<*0.05*	*0.078*	*7.8* %
	*PBL Evaluation Tutorial Process and COMPASS*® *generic competencies*	*0.315*–*0.407*	<*0.01*	*0.099*–*0.166*	*9.9*–*16.6* %
	*PBL Evaluation Reflective Journal and COMPASS*® *Occupational competencies overall score*	*0.271*	<*0.05*	*0.073*	*7.3* %
	*PBL Evaluation Tutorial Process and COMPASS*® *Occupational competencies overall score*	*0.367*	<*0.01*	*0.135*	*13.5* %
Kahn et al. [29] Medicine	*OSCE and Program director evaluations*	*0.22*	*0.15*	0.048	4.8%
McLaughlin et al. [33] Pharmacy	*Year 2 Fall OSCE and APPE online evaluations*	*0.13*–*0.14*	<*0.05*	*0.017*–*0.020*	*1.7*–*2* %
	*Year 2 Spring OSCE and Acute Care APPE*	*0.12*	<*0.05*	*0.014*	*1.4* %
	*Year 3 OSCE and*:				
	- *acute care APPE*	*0.12*	<*0.05*	*0.014*	*1.4* %
	- *ambulatory care APPE*	*0.25*	<*0.01*	*0.063*	*6.3* %
	- *clinical specialty APPE*	*0.13*	<*0.05*	*0.017*	*1.7* %
Wessel et al. [31] Physiotherapy	*OSCE and Physiotherapy Clinical Performance Instrument*	−*0.13*	*Reported as not significant*	0.017	1.7%
Wilkinson & Frampton [21] Medicine	*OSCE and Global rating instrument*				
	- *total score*	*0.59*	<*0.001*	*0.348*	*34.8* %
	- *clinical skills subscale*	*0.63*	<*0.001*	*0.397*	*39.7* %
	- *Humanistic subscale*	*0.44*	<*0.001*	*0.194*	*19.4* %
	*Written 1* (*3* × *3 h short and long essay questions*) *and Global rating instrument*				
	- *total score*	*0.17*	*0.201*	*0.029*	*2.9* %
	- *clinical skills subscale*	*0.24*	*0.071*	*0.058*	*5.8* %
	- *humanistic subscale*	*0.05*	*0.738*	*0.003*	*0.03* %
	*Written 2* (*1* × *3 h short essay and 2* × *3 h EMQ*) *and Global rating instrument*				
	- *total score*	*0.54*	<*0.001*	*0.292*	*29.2* %
	- *clinical skills subscale*	*0.57*	<*0.001*	*0.325*	*32.5* %
	- *humanistic subscale*	*0.41*	<*0.001*	*0.168*	*16.8* %

*APPE* advanced pharmacy practice experiences, *CIS* communication and Interpersonal Skills, *COMLEX*-*USA Level 2*-*PE* Comprehensive Osteopathic Medical Licensing Examination of the United States Level 2-Performance Evaluation, *CPX* Clinical Performance examination, *CRDTS* Central Region Dental Testing Service, *EMQ* Extended Matching Question, *HKU* Hong Kong University, *ICE* integrated clinical encounter; *JDAT* Junior Doctor Assessment Tool, *MCQ* multiple choice question, *OSCE* objective Structured Clinical Examination, *PBL* problem based learning, *PT CPI* physiotherapy clinical performance instrument, *SAQ* short answer question, *USMLE Step 2 CS* United States Medical Licensing Examination Step 2 Clinical Skills

### Objective structured clinical examination

Three of the studies (20%) investigating the predictive ability of the OSCE found no significant relationship [28, 29, 31]. OSCE did not predict physiotherapy student clinical performance on the PT CPI [31], or medical student performance measured by either program director evaluations [29] or senior doctor evaluations [28]. Nine of twelve studies in the medical profession (75%) identified a significant positive relationship between medical student OSCE scores and clinical performance [19–27, 30], with OSCE scores explaining between 1.9 and 39.7% of the variability in medical student clinical performance. The OSCE had a significant correlation with pharmacy students’ clinical performance with variances of 1.4–6.3% [33]. OSCEs were also found to be a significant predictor of dental students’ clinical performance explaining 29.2–37.7% of the variability in clinical productivity values [34]. A significant relationship was reported between pre-clinical OSCE scores and the clinical performance of dietetic students (β = 0.66; 95% CI 0.46–0.86; *P* < 0.0001) [32].

### Written examinations

Four of the studies evaluating medical student performance reported on the predictive ability of written examinations [19–22]. Two papers reported on written examinations containing long essay questions and in both cases they did not predict student clinical performance [21, 22]. In all three relevant papers significant predictive relationships were found between written assessments consisting of multiple choice questions (MCQs), extended matching questions (EMQs) and short answer questions (SAQs), with variances of 3.2, 7.3 and 29.2% [19–21].

### Other assessments

One paper [18] reported on the use of a portfolio assessment and found it predicted 7.3% of the variability in dental hygiene student clinical performance. A PBL evaluation consisting of three assessment items predicted 5.9–16.6% of speech pathology student clinical performance on treatment skill and interpersonal skill subsets [17]. Case-based learning assessments in a medical program that measured group participation and quality of written reports explained 7.3 and 4.8% of the variance students clinical performance respectively [19].

### Prediction models

A prediction model for medical student clinical performance incorporating Year 4 and 5 OSCEs, Year 5 and 6 written examinations, scores from Year 6 clinical attachments and overall GPA identified that no individual summative assessment significantly influenced the clinical performance score; the best overall predictor of clinical performance measured by the JDAT was overall GPA [20]. A second paper [21] combined the OSCE and written examination results of medical students in a multiple regression model and found that the OSCE added significantly to the correlation with clinical performance scores. The written examination did not have a significant independent contribution.

## Discussion

The aim of this review was to critically appraise and discuss the findings of existing research investigating the ability of summative assessments used within the non-clinical components of an academic curriculum to predict clinical performance across the breadth of health profession education. Eighteen studies that met inclusion and exclusion criteria were critically reviewed. The overall methodological quality of the literature that was investigated to inform this review was considered to be ‘fair’. None of the studies included in the review were found to report on: (i) the principle confounders, (ii) the power of the research and (iii) attempts to blind either participants or those measuring clinical performance. The studies that scored more highly clearly described the summative assessment being investigated and the main findings, as well as reported actual probability values and the characteristics of students lost to follow up.

The OSCE is well established in health education programs worldwide. It is a mode of assessment specifically designed to provide a valid and reliable measure of students’ clinical competence in a simulated environment [11]. Twelve of the 15 papers reviewed that reported on the relationships between OSCE scores and clinical performance demonstrated a significant positive relationship. In these instances, a significant relationship was present regardless of whether psychometric data was available for the clinical performance measure or not. Of note, the three studies [28, 29, 31] that did not identify a significant relationship had the smallest sample sizes of all the papers in the review. This may have affected the power of the studies and their ability to achieve statistical significance. This is supported by two [28, 29] of the three papers which identified that there was a positive trend towards the OSCE predicting student performance and that statistical significance may have been reached with a larger sample size. The clinical performance measures used by studies included in this review assessed similar domains of competency to OSCEs, although in more complex and often less structured environments. OSCEs assess student performance at the ‘shows how’ level of Miller’s pyramid [36]; it is likely that the clinical performance measures also evaluate students at the ‘shows how’ level as there is a strong argument that ‘does’ can only be measured when the candidate is unaware of being observed or assessed [37]. The similarities between both the domains of competence and the levels of performance measured provides some explanation for the consistent positive relationship reported between students OSCE scores and their future clinical performance.

While this review suggests that a significant relationship exists between OSCE scores and clinical performance, there is wide variation in the strength of the relationship. With the OSCE explaining between 1.9% [20] and 39.7% [21] of the variation in student clinical performance, the strength of the relationships may have been influenced by other factors that in turn may vary between programs. One such factor is the structure of the OSCE itself. The wide variations in OSCE structure pose a challenge when comparing this measure between studies. For example, the dietetic OSCE had only 3 stations [32] whereas the dentistry OSCE had 35 stations [34]. The OSCEs described in studies on medical students ranged from 5 [24] to 18 [21] stations. The papers with the two strongest predictive relationships between OSCE and student clinical performance described OSCEs with 18 × 5 min stations [21] and 35 × 2 min stations [34] which suggests that longer OSCE assessments may be better predictors of performance. This finding is supported by a systematic review [38] of the reliability of the OSCE in medical education programs which identified that while scores on OSCEs are not always very reliable, better reliability was associated with a greater number of stations. This is attributed to a wider sampling of cases across the increased number of stations. Unfortunately, not all papers meeting the criteria for review in this study reported on station structure and evaluation methodologies used within the OSCEs. This limited the ability to further discuss the impact of OSCE structure on the predictive ability of the assessment but may explain the large differences in variance.

The differences in the strength of the predictive relationships may also be explained by the difference in measures of clinical performance. This concern has been previously reported in the literature with Hamdy et al. [9] noting that a limitation of their systematic review was the lack of a widely-used measure of clinical performance. The findings of the present review also need to be considered in light of the limitations imposed by the variety of clinical performance measures used.

A variance of 1.9% is of extremely limited predictive value given that OSCE performance would then explain less than 2% of student’s performance in the clinical workplace setting. However, a variance of 37.7% indicates a strong predictive relationship. A predictive relationship of this strength would be valuable for assisting to identify students at risk of poor performance in the clinical setting. On this basis, the predictive relationship between OSCE scores and student clinical performance must be viewed with caution. However, these scores could be used by educators as a method of identifying students that may be at risk of low performance in a clinical practice setting until a more robust measure is available.

As only one paper was identified for each of the portfolio, case-based and problem-based learning assessments there is inadequate data to draw conclusions about these modes of assessment. Four papers in the review did investigate written assessments. Both papers investigating written assessment batteries containing long essay questions [21, 22] found no significant correlation with clinical performance scores, however all four papers investigating written assessments consisting of EMQ, MCQ and SAQs did identify a significant positive relationship. This supports literature advocating the use of EMQs or MCQs in written examinations rather than essay questions [39]. Like the findings for the OSCE, there was a large difference in the strength of the relationship between papers reviewed. An EMQ/MCQ written assessment explained 29.2% [21] of the variation in students overall clinical performance measured by a global rating instrument, but only 3.2% [20] when clinical performance was measured by the JDAT. While other program factors other than the choice of clinical performance measure may also influence these relationships, there is a large difference in the ability of the MCE/EMQ written assessments to predict clinical performance. This highlights the need for research to occur where a standard measure of clinical performance is used to allow for comparison between studies. The findings of this review suggest that there is limited evidence to support the use of SMQ, MCQ and EMQ written assessments to predict student’s clinical performance and that the written examinations should be used as a predictive measure with caution.

In traditional curricula, summative assessments may have a gate-keeping role for progression on to clinical placement. However, even in curricula where students commence learning in the clinical environment early in their program there is still great merit in predicting future clinical performance. The early identification of students at risk of poor performance allows for targeted remediation prior to clinical experiences, as well as the implementation of focused support whilst the student is embedded in the clinical environment. However, until further research adds to the body of evidence, the use of summative assessments to predict student clinical performance should be approached with caution. If educators choose to use summative assessment results to attempt to predict clinical performance then this review suggests that the OSCE, which has a weak predictive value, may be the most appropriate choice. This review also implies that individual modes of summative assessment should not be the gatekeepers into the clinical practice environment as there is insufficient evidence to base high-stakes decisions (such as a student’s ability to progress on to clinical placement) on the predictive ability of these assessments.

In addition to the differences in the structure of summative assessments investigated and clinical performance measures used that this review has already discussed, a potential limitation of the research reviewed is that only students who completed their program of study were included. Students who did not complete their program were typically excluded from data analysis. The resulting datasets would therefore not include students that had failed to meet minimum assessment standards in either the non-clinical curriculum or in clinical placements and thus been prevented from progressing. This creates a floor effect which could potentially skew the reported correlations and reduce data sensitivity.

Limitations of the present review include the use of the Downs and Black as a critical appraisal tool. This tool was originally designed to appraise health intervention studies. While it has enabled a standardised critique of the studies in this review, it may be that the papers have been appraised more harshly when applied to the same critique as an interventional study. Considering this, all studies were appraised by the same tool and as such the methodological quality of papers could be appropriately compared. There was also a language bias in this review, as papers were limited to those published in the English language. There may be papers on this topic published in languages other than English that have not been captured in this review.

Future research on this topic should aim to recruit larger sample sizes to increase statistical power. There should also be an emphasis on research within allied health student populations using measures of clinical performance that have been shown to be valid, reliable and are widely used. This approach would allow for a more rigorous comparison between programs and even professions to be conducted, aiding in the generalisation of findings across the allied health professions.

## Conclusion

The findings of this review suggest that assessments used within an academic curriculum do have significant positive relationships with the clinical performance of health professional students. To use these assessments as predictive measures caution is required due to a small body of evidence and large variations in the predictive strength of the relationships identified. The OSCE may be the most appropriate choice at this time for educators planning to use summative assessment scores to identify students that may be at risk of poor performance in a clinical workplace environment. Further research, with larger sample sizes, is required to determine the ability of summative assessments to predict the future clinical performance of health profession students particularly in allied health student populations.

## Additional file

Additional file 1: Papers excluded from review and reasons for exclusion. (DOCX 30 kb)

## Abbreviations

APPE: Advanced Pharmacy Practice Experiences
COMLEX-USA Level 2-PE: Comprehensive Osteopathic Medical Licensing Examination of the United States Level 2 Performance Evaluation
CPX: Clinical performance examination
CRDTS: Central Region Dental Testing Service
EMQ: Extended matching question
GPA: Grade point average
HKU: Hong Kong University
JDAT: Junior Doctor Assessment Tool
MCQ: Multiple choice question
NDHE: National Dental Hygiene Examination
OSCE: Objective structured clinical examination
PBL: Problem-based learning
PT CPI: Physiotherapy Clinical Performance Instrument
SAQ’s: Short answer questions
COMPASS®: Competency Based Assessment in Speech Pathology
USMLE Step 2 CS: United States Medical Licensing Examination Step 2 Clinical Skills
