# Supplementary material for: Do coursework summative assessments predict clinical performance? A systematic review
Source: BMC Med Educ. 2017 Feb 16;17:40. doi: 10.1186/s12909-017-0878-3 (PMC5314623; doi:10.1186/s12909-017-0878-3)
Supplement: Additional file 1: — Papers excluded from review and reasons for exclusion. (DOCX 30 kb) [file 12909_2017_878_MOESM1_ESM.docx]

# Do coursework summative assessments predict clinical performance? A systematic review.

# Additional File 1 – Studies excluded from critical review and reasons for exclusion

### Exclusion Criteria

1. The independent variable is a formative assessment
2. Individual coursework summative assessments methods were not specified (e.g. used overall grade-point average)
3. The independent variable was a standardised assessment limited to use by a single health profession (e.g. National Board of Medical Examiners subject examinations)
4. The independent variables were health profession education program admission criteria, applicant screening measures or entry measures
5. Studies did not measure the dependent variable clinical performance in either a clinical workplace setting or in a clinical examination conducted externally to the education program utilising real or standardized patients
6. Paper was an abstract, review, dissertation or discussion

| Reference | Reason for exclusion |
| --- | --- |
| Alexander GL, Davis WK, Yan AC, Fantone III JC. Following medical school graduates into practice: residency directors' assessments after the first year of residency. Acad Med. 2000;75(10):S15-S17. | B |
| Andonian L. Emotional intelligence, self-efficacy, and occupational therapy students' fieldwork performance. Occup Ther Health Care. 2013;27(3):201-215. | A |
| Andriole DA, Jeffe DB, Whelan AJ. What predicts surgical internship performance? Am J Surg. 2004;188(2):161-4. | B |
| Amos DE, Massagli TL. Medical school achievements as predictors of performance in a physical medicine and rehabilitation residency. Acad Med. 1996;71(6):678. | B |
| Artino AR, Gillilan WR, Waechter DM, Cruess D, Calloway M, Durning SJ. Does self-reported clinical experience predict performance in medical school and internship? Med Educ. 2012;46(2):172-8. | D |
| Baggs T, Barnett D, McCullough K. The value of traditional cognitive variables for predicting performance in graduate Speech-Language Pathology programs. J Allied Health. 2015;44(1):10-6. | B, D |
| Basco WT, Jr Gilbert GE, Chessman AW, Blue AV. The ability of a medical school admission process to predict clinical performance and patients' satisfaction. Acad Med. 2000;75(7):743-7. | D |
| Beauvais AM, Brady N, O'Shea ER, Griffin MT. Emotional intelligence and nursing performance among nursing students. Nurse Educ Today. 2011;31(4):396-401. | A |
| Bolender JS. Predictors of certification scores in family nurse practitioners: personality, academic, and demographic factors. Cardinal Stritch University. 2001. | F |
| Boyse TD, Patterson SK, Cohan RH, Korobkin M, Fitzgerald JT, Oh MS, Quint DJ. Does medical school performance predict radiology resident performance? Acad Radiol. 2002;9(4):437-45. | B, C |
| Brailovsky C, Charlin B, Beausoleil S, Coté S, Van der Vleuten C. Measurement of clinical reflective capacity early in training as a predictor of clinical reasoning performance at the end of residency: an experimental study on the script concordance test. Med Educ. 2001;35(5):430-6. | E |
| Brown G, Imel B, Nelson A, Hale LS, Jansen N. Correlations between PANCE performance, physician assistant program grade point average, and selection criteria. J Physician Assist Educ. 2013;24(1):42-4. | B |
| Burish MJ, Fredericks CA, Engstrom JW, Tateo VL, Josephson SA. Predicting success: What medical student measures predict resident performance in neurology? Clin Neurol Neurosurg. 2015;135:69-72. | B, C, E |
| Callahan CA, Hojat M, Veloski J, Erdmann JB, Gonnella JS. The predictive validity of three versions of the MCAT in relation to performance in medical school, residency, and licensing examinations: a longitudinal study of 36 classes of Jefferson Medical College. Acad Med. 2010:85(6):980-7. | D |
| Carpio B, O'Mara L, Hezekiah J. Predictors of success on the Canadian Nurses Association testing service (CNATS) examination. Can J Nurs Res. 1996;28(4):115-23. | B |
| Corcoran J, Halverson A L, Schindler N. A formative midterm test increases accuracy of identifying students at risk of failing a third year surgery clerkship. Am J Surg. 2014;207(2):260-2. | A |
| Curtis DA, Lind SL, Brear S, Finzen FC. The correlation of student performance in preclinical and clinical prosthodontic assessments. J Dent Educ. 2007;71(3):365-72. | E |
| Danielson JA, Wu TF, Molgaard LK, Preast VA. Relationships among common measures of student performance and scores on the North American Veterinary Licensing Examination. J Am Vet Med Assoc. 2011;238(4):454-61. | E |
| Denton GD, Durning SJ, Wimmer AP, Pangaro LN, Hemmer PA. Is a faculty developed pretest equivalent to pre-third year GPA or USMLE step 1 as a predictor of third-year internal medicine clerkship outcomes? Teach Learn Med. 2004;16(4):329-32. | A |
| Dixon D. Prediction of Osteopathic Medical School Performance on the basis of MCAT score, GPA, sex, undergraduate major, and undergraduate institution. J Am Osteopath Assoc. 2012: 112(4):175-81. | B, D |
| Dong T, Saguil A, Artino AR, Jr., Gilliland WR, Waechter DM, Lopreaito J, Flanagan A, Durning SJ. Relationship between OSCE scores and other typical medical school performance indicators: a 5-year cohort study. Mil Med. 2012;177(9 Suppl):44-6. | E |
| Edgar S, Mercer A, Hamer P. Admission interview scores are associated with clinical performance in an undergraduate physiotherapy course: an observational study. Physiotherapy. 2014;12(14). | D |
| Efurd MG. Predictors of Academic Success for the National Board Dental Hygiene Examination and the Southern Regional Testing Agency Clinical Exam. ProQuest LLC, Michigan. 2012. | F |
| Elliott MJ. Academic Predictors of National Council Licensure Examination for Registered Nurses Pass Rates. Walden University. 2011. | F |
| Ennulat CW, Garrubba C, DeLong D. Evaluation of multiple variables predicting the likelihood of passage and failure of PANCE. J Physician Assist Educ. 2011;22(1):7-18. | E |
| Evans P, Goodson LB, Schoffman SI. Relationship between academic achievement and student performance on the Comprehensive Osteopathic Medical Licensing Examination-USA level 2. J Am Osteopath Assoc. 2003;103(7):331-6. | B |
| Evans P, Goodson LB, Schoffman SI, Baker HH. Relations between academic performance by medical students and COMLEX-USA Level 2: a multisite analysis. J Am Osteopath Assoc. 2003;103(11):551-6. | B |
| George AB, Schuster A, Helmer SD, Drake RM, Silkey B, Cusick TE, Osland JS, Ammar AD. Do medical student's surgical examination scores correlate with performance markers? Am J Surg. 2014;208(6):1040-6. | E |
| Gonnella JS, Erdmann JB, Hojat M. An empirical study of the predictive validity of number grades in medical school using 3 decades of longitudinal data: implications for a grading system. Med Educ. 2004;38(4):425-34 | B |
| Greenburg DL, Durning SJ, Cohen DL, Cruess D, Jackson JL. Identifying medical students likely to exhibit poor professionalism and knowledge during internship. J Gen Intern Med. 2007;22(12):1711-7. | B |
| Hamdy H, Prasad K, Anderson MB, Scherpbier A, Williams R, Zwierstra R, Cuddihy H. BEME systematic review: predictive values of measurements obtained in medical schools and future performance in medical practice. Med Teach. 2006;28(2):103-16. | F |
| Harfmann KL, Zirwas MJ. Can performance in medical school predict performance in residency? A compilation and review of correlative studies. J Am Acad Dermatol. 2011;65(5):1010-22. | F |
| Hojat M, Gonnella JS, Mangione S, Nasca TJ, Veloski JJ, Erdmann JB, Callahan CA, Magee M. Empathy in medical students as related to academic performance, clinical competence and gender. Med Educ. 2002;36(6):522-7. | A, E |
| Howard L, Jerosch-Herold C. Can entry qualifications be used to predict fieldwork and academic outcomes in occupational therapy and physiotherapy students? The British Journal of Occupational Therapy. 2000;63(7):329-34. | D |
| Hu Y, Martindale JR, LeGallo RD, White CB, McGahren ED, Schroen AT. Relationships between preclinical course grades and standardized exam performance. Adv Health Sci Educ Theory Pract. 2016;21(2):389-99. | B |
| Inda KC. Relationship between clinical reasoning skills and certification exam performance in occupational therapy candidates. Nova Southeastern University. 2007. | F |
| Kirchner GL & Holm MB. Prediction of academic and clinical performance of occupational therapy students in an entry-level master's program. Am J Occup Ther. 1997;51(9):775-9. | D |
| Kirchner GL, Stone RG, Holm MB. Use of Admission Criteria to Predict Performance of Students in an Entry-Level Master's Program on Fieldwork Placements and in Academic Courses. Occup Ther Health Care. 2001;13(1):1-10. | D |
| Kosmahl EM. Factors related to physical therapist license examination scores. Journal of Physical Therapy Education. 2005;19(2):52-6. | E |
| Kulatunga-Moruzi C, & Norman GR. Validity of admissions measures in predicting performance outcomes: the contribution of cognitive and non-cognitive dimensions. Teach Learn Med. 2002;14(1):34-42. | D |
| Lawson DM, Till H. Predictors of performance of students from the Canadian Memorial Chiropractic College on the licensure examinations of the Canadian Chiropractic Examining Board. J Manipulative Physiol Ther. 2006;29(7):566-69. | E |
| Lewis ES. A study of emotional intelligence, cognitive intelligence and clinical performance of physical therapy students. University of Massachusetts Lowell. 2004. | F |
| Lewis E. Emotional intelligence as a predictor for clinical performance in professional physical therapy students. Internet Journal of Allied Health Sciences & Practice. 2010;8(4):1-8. | A |
| Lievens F, Sackett PR. The validity of interpersonal skills assessment via situational judgment tests for predicting academic success and job performance. J Appl Psychol. 2012;97(2):460-8. | D |
| Luedtke-Hoffmann K, Dillon L, Utsey C, Tomaka J. Is there a relationship between performance during physical therapist clinical education and scores on the National Physical Therapy Examination (NPTE)? Journal of Physical Therapy Education. 2012;26(2):41-9. | B, C, E |
| Lurie SJ, Lambert DR, Nofziger AC, Epstein RM, Grady-Weliky TA. Relationship between peer assessment during medical school, dean's letter rankings, and ratings by internship directors. J Gen Intern Med. 2007;22(1):13-6. | A |
| Martin IG & Jolly B. Predictive validity and estimated cut score of an objective structured clinical examination (OSCE) used as an assessment of clinical skills at the end of the first clinical year. Med Educ. 2002;36(5):418-25. | E |
| Massey SL, Lee L, Young S, Holmerud D. The relationship between formative and summative examination and PANCE results: a multi-program study. J Physician Assist Educ. 2013;24(1):24-34. | B, E |
| McCauley DT. Predictors of Canadian registered nurse examination performance: Nursing coursework, clinical courses, and length to program completion. D'Youville College. 2014. | F |
| Merrick HW, Nowacek G, Boyer J, Robertson J. Comparison of the objective structured clinical examination with the performance of third-year medical students in surgery. Am J Surg 2000;179(4):286-8. | A |
| Middlemas DA, Manning JM, Gazzillo LM, Young J. Predicting Performance on the National Athletic Trainers' Association Board of Certification Examination From Grade Point Average and Number of Clinical Hours. J Athl Train. 2001;36(2):136-40. | B, E |
| Morris J & Farmer A. The predictive strength of entry grades and biographical factors on the academic and clinical performance of physiotherapy students. Physiother Theory Pract. 1999;15(3):165-73. | D |
| Morrison CA, Ross LP, Sample L, Butler A. Relationship between performance on the NBME(R) Comprehensive Clinical Science Self-Assessment and USMLE(R) Step 2 Clinical Knowledge for USMGs and IMGs. Teach Learn Med. 2014;26(4):373-8. | A, C, E |
| Muller ES, Harik P, Margolis M, Clauser B, Mckinley D, Boulet JR. An examination of the relationship between clinical skills examination performance and performance on USMLE Step 2. Acad Med. 2003:78(10):S27-9. | B |
| Nadasan T & Puckree T. Do the selection criteria for admittance to the physiotherapy program predict students' performance? South African Journal of Physiotherapy. 2003;59(3):20. | D |
| Nelson LP, Maramaldi P, Kinnunen TH, Kalenderian E. Early performance in a humanistic medicine course as a predictor of dental students' later clinical performance. J Dent Educ. 2013;77(8):1006-12. | B |
| Nunez DW, Taleghani M, Wathen WF, Abdellatif HM. Typodont versus live patient: predicting dental students' clinical performance. J Dent Educ. 2012;76(4):407-13. | E |
| Park SE, Susarla SM, Massey W. Do admissions data and NBDE Part I scores predict clinical performance among dental students? J Dent Educ. 2006;70(5):518-24. | C, D |
| Pearson SA, Rolfe IE, Henry RL. The relationship between assessment measures at Newcastle Medical School (Australia) and performance ratings during internship. Med Educ. 1998;32(1):40-5. | B |
| Pepple DJ, Young LE, Gordon-Strachan GM, Carroll RG. Pre-clinical grades predict clinical performance in the MBBS stage II examination at the University of the West Indies, Mona Campus. Niger J Physiol Sci. 2013;28(2)201-4. | B, E |
| Peskun C, Detsky A, Shandling M. Effectiveness of medical school admissions criteria in predicting residency ranking four years later. Med Educ. 2007;41(1):57-64. | D |
| Ranney RR, Gunsolley JC, Miller LS, Wood M. The relationship between performance in a dental school and performance on a clinical examination for licensure: a nine-year study. J Am Dent Assoc. 2004;135(8):1146-53. | B |
| Rice EW. The relationship between emotional intelligence, self-efficacy, and clinical performance in associate degree nursing students. Capella University. 2013. | A |
| Ripkey DR, Case SM, Swanson DB. Identifying students at risk for poor performance on the USMLE Step 2. Acad Med. 1999;74(10 Suppl):S45-8. | C, E |
| Roberts CM. Relationships among admission variables, professional education outcome measures, and job performance of University of Missouri physical therapy graduates. University of Missouri, Columbia. 1996. | F |
| Roberts WL, Pugliano G, Langenau E, Boulet JR. Modeling relationships between traditional preadmission measures and clinical skills performance on a medical licensure examination. Adv Health Sci Educ Theory Pract. 2012;17(3):403-17. | D |
| Roth KS, Riley WT, Brandt RB, Seibel HR. Prediction of students' USMLE step 2 performances based on premedical credentials related to verbal skills. Acad Med. 1996;71(2);176-80. | B, E |
| Saguil A, Dong T, Gingerich RJ, Swygert K, LaRochelle JS, Artino Jr AR, Cruess DF, Durning SJ. Does the MCAT Predict Medical School and PGY-1 Performance? Mil Med. 2015;180(4):4-11. | D |
| Sandow PL, Jones AC, Peek CW, Courts FJ, Watson RE. Correlation of admission criteria with dental school performance and attrition. J Dent Educ. 2002;66(3):385-92. | D,E |
| Sawhill A, Butler A, Ripkey D, Swanson DB, Subhiyah R, Thelman J, Angelucci K. Using the NBME self-assessments to project performance on USMLE Step 1 and Step 2: impact of test administration conditions. Acad Med. 2004;79 Suppl 10:55-57. | A, C, E |
| Scior K, Bradley CE, Potts HW, Woolf K, & de CWAC. What predicts performance during clinical psychology training? Br J Clin Psych. 2014;53(2):194-212. | D |
| Scott JN, Markert RJ, Dunn MM. Critical thinking: change during medical school and relationship to performance in clinical clerkships. Med Educ. 1998;32(1):14-8. | A |
| Shelledy DC, Gardner DD, Carpenter ME, Murphy DL. The relationship between general critical thinking ability and student performance. Respiratory Care Education Annual. 2004;13:23-8. | A, B |
| Silver B & Hodgson CS. Evaluating GPAs and MCAT scores as predictors of NBME I and clerkship performances based on students' data from one undergraduate institution. Acad Med. 1997;72(5):394-6. | B |
| Simon SR, Bui A, Day S, Berti D, Volkan K. The relationship between second-year medical students' OSCE scores and USMLE Step 2 scores. J Eval Clin Pract. 2007;13(6):901-5. | E |
| Sisola SW. Moral reasoning as a predictor of clinical practice: the development of physical therapy students across the professional curriculum. Journal of Physical Therapy Education. 2000;14(3):26-34. | A |
| Stacey DG & Whittaker JM Predicting academic performance and clinical competency for international dental students: seeking the most efficient and effective measures. J Dent Educ. 2005;69(2):270-80. | D |
| Stewart CM, Bates RE, Jr., Smith GE. Relationship between performance in dental school and performance on a dental licensure examination: an eight-year study. J Dent Educ. 2005;69(8):864-9. | B |
| Tan K, Meredith P, McKenna K. Predictors of occupational therapy students' clinical performance: an exploratory study. Australian Occupational Therapy Journal. 2004;51(1):25-33. | A,D |
| Thieman TJ, Weddle ML, Moore MA. Predicting academic, clinical, and licensure examination performance in a professional (entry-level) master's degree program in physical therapy. Journal of Physical Therapy Education. 2003;17(2):32-7. | D |
| Tomlin G. The use of interactive video client simulation scores to predict clinical performance of occupational therapy students. Am J Occup Ther. 2005;59(1):50-6. | B |
| Townsend AH, McLlvenny S, Miller CJ, Dunn EV. The use of an objective structured clinical examination (OSCE) for formative and summative assessment in a general practice clinical attachment and its relationship to final medical school examination performance. Med Educ. 2001;35(9):841-6. | E |
| Velayo BC, Stark PC, Eisen SE, Kugel G. Using dental students' preclinical performance as an indicator of clinical success. J Dent Educ. 2014;78(6):823-8. | E |
| Vendrely AM. An investigation of the relationships between academic performance, clinical performance, critical thinking, and success on the physical therapy education program. Loyola University of Chicago. 2002. | A, B, F |
| Victoroff KZ & Boyatzis RE. What is the relationship between emotional intelligence and dental student clinical performance? J Dent Educ. 2013;77(4):416-26. | A |
| Wass V, Van Der Vleuten C, Shatzer J, Jones R. Assessment of clinical competence. Lancet. 2001;357(9260):945-9. | F |
| Watson CJ, Barnes CA, Williamson JW. Determinants of clinical performance in a physical therapy program. J Allied Health. 2000;29(3):150-6. | D |
| Wettstein RB, Wilkins RL, Gardner DD, Restrepo RD. Critical-thinking ability in respiratory care students and its correlation with age, educational background, and performance on national board examinations. Respir Care. 2011;56(3):284-9. | A |
| White CB, Dey EL, Fantone JC. Analysis of factors that predict clinical performance in medical school. Adv Health Sci Educ Theory Pract. 2009;14(4):455-64. | D |
| Wiggers TB, Holton RH. Predictive value of a senior comprehensive examination as to performance on a national certification examination. Clin Lab Sci. 2001;14(1):21-6. | E |
| Williams KB, Glasnapp DR, Tilliss TS, Osborn J, Wilkins K, Mitchell S, Kershbaum W, Schmidt C. Predictive validity of critical thinking skills for initial clinical dental hygiene performance. J Dent Educ. 2003;67(11):1180-92. | A, E |
| Williams RG, Klamen DL. Twenty Questions game performance on medical school entrance predicts clinical performance. Med Educ. 2015;49(9):920-7. | A |
| Williams KB, Schmidt C, Tilliss TS, Wilkins K, Glasnapp DR. Predictive validity of critical thinking skills and disposition for the national board dental hygiene examination: a preliminary investigation. J Dent Educ. 2006;70(5):536-44. | A |
| Woloschuk W, McLaughlin K, Wright B. Is undergraduate performance predictive of postgraduate performance? Teach Learn Med. 2010;22(3):202-4. | B |
| Yoho RM, Tallerico V, Vardaxis V. Relationship between student academic and clinical performance in podiatric medical education at Des Moines University. J Am Podiatr Med Assoc. 2012;102(4):314-8. | B |
